# Publisher Correction: Few keystone plant genera support the majority of Lepidoptera species

**DOI:** 10.1038/s41467-021-21304-2

**Published:** 2021-02-10

**Authors:** Desiree L. Narango, Douglas W. Tallamy, Kimberley J. Shropshire

**Affiliations:** 1 Department of Entomology and Wildlife Ecology, University of Delaware, Newark, DE 19716 USA; 2 Present Address: Department of Biology, University of Massachusetts, Amherst, MA 1002 USA

**Keywords:** Biodiversity, Food webs, Conservation biology, Ecological networks

Correction to: *Nature Communications* 10.1038/s41467-020-19565-4, published online 13 November 2020.

The original version of this Article contained errors in Fig. 3, its associated legend, and one of its references in the main text.

First, headings were cut off from the top of the figure. The correct version of Fig. 3 is: 

Which replaces the previous incorrect version:

The correct legend for Fig. 3 is:

“Fig. 3 including woody and herbaceous keystone plants increases the efficacy and efficiency of the restoration of Lepidoptera diversity. Simulations of restoration scenarios where (a) Lepidoptera diversity on woody plants, (b) Lepidoptera diversity on herbaceous plants, (c) interaction diversity on woody plants, and (d) interaction diversity on herbaceous plants as numbers of plant genera (*x*-axis) and keystone plant genera (*y*-axis) increase. For comparisons across counties, percent species and percent interactions from total possible is used in lieu of raw numbers. For both woody (left column) and herbaceous plants (right column), supported caterpillar species and interactions were higher with fewer plants when keystone plant species were included. In woody plants, more than twice the number of plant genera was required to achieve the species and interactions produced by keystone plants. In herbaceous plants, comparable richness and interactions were not reached even with a 3-fold increase in plant genera richness. Caterpillar icon in (a, b) from www.phylopic.org; caterpillar and leaf icons in (c, d) made by Freepik [https://www.flaticon.com/authors/freepik] from www.flaticon.com.”

Which replaces the previous incorrect version:

“Fig. 3: Including woody keystone plants increases the efficacy and efficiency of the restoration of Lepidoptera diversity. Simulations of restoration scenarios where (a, b) Lepidoptera diversity and (c, d) interaction diversity as numbers of plant genera (*x*-axis) and keystone plant genera (*y*-axis) increase in woody and herbaceous plants. For comparisons across counties, percent species and percent interactions from total possible is used in lieu of raw numbers. For both woody and herbaceous plants, supported caterpillar species and interactions were higher with fewer plants when keystone plant species were included. In woody plants, more than twice the number of plant genera was required to achieve the species and interactions produced by keystone plants. In herbaceous plants, comparable richness and interactions were not reached even with a 3-fold increase in plant genera richness. Caterpillar icon in (*a*, *b*) from www.phylopic.org; caterpillar and leaf icons in (*c*, *d*) made by Freepik [https://www.flaticon.com/authors/freepik] from www.flaticon.com.”

Finally, the original version of the Article contained a sentence with a reference to Fig. 4, which does not exist because it was merged with Fig. 3 during production: “Our simulation revealed that restored Lepidoptera and interaction diversity could be nearly doubled by the presence of just a few productive taxa for both woody (Fig. 3) and herbaceous plants (Fig. 4).” This incorrect sentence has been replaced with “Our simulation revealed that restored Lepidoptera and interaction diversity could be nearly doubled by the presence of just a few productive taxa for both woody and herbaceous plants (Fig. 3).”

These errors have now been corrected in both the PDF and HTML versions of the Article.

