# Supplementary material for: Few keystone plant genera support the majority of Lepidoptera species
Source: Nat Commun. 2020 Nov 13;11:5751. doi: 10.1038/s41467-020-19565-4 (PMC7666120; doi:10.1038/s41467-020-19565-4)
Supplement: Supplementary file 1 — Supplementary Information [file 41467_2020_19565_MOESM1_ESM.pdf]

Supplementary Materials for

FEW KEYSTONE PLANT GENERA SUPPORT THE MAJORITY OF LEPIDOPTERA SPECIES

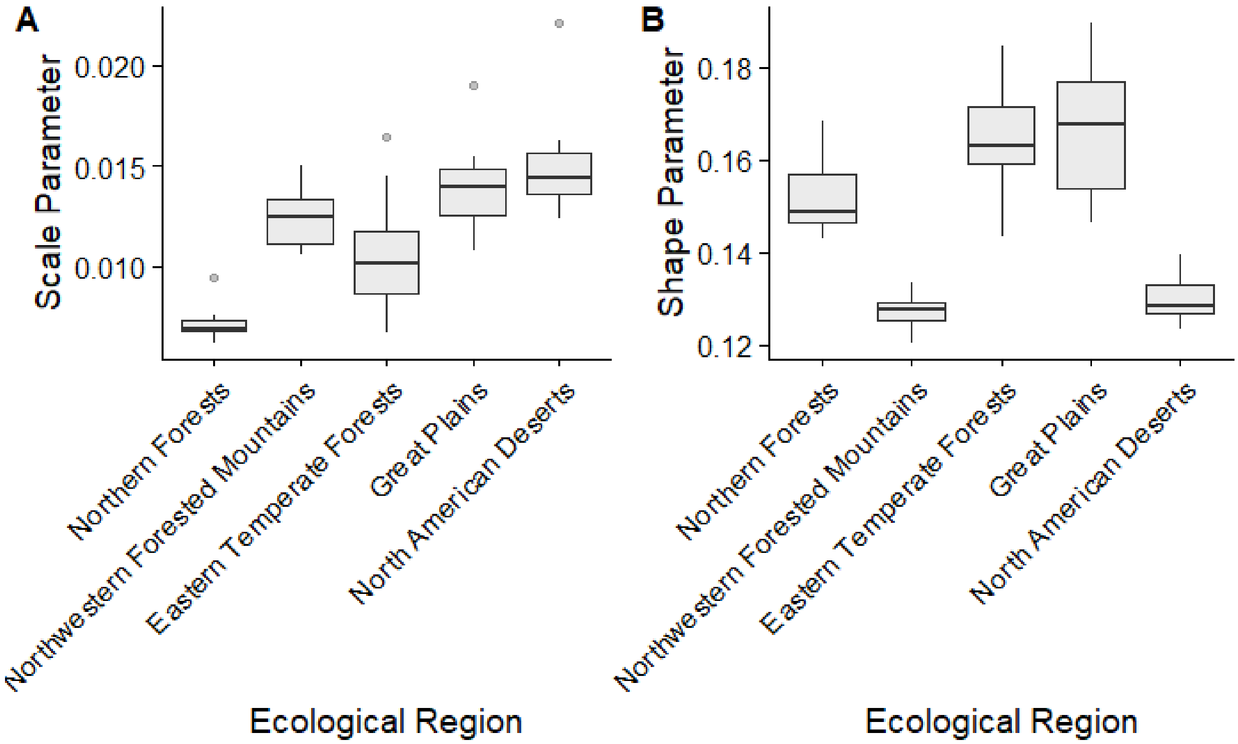

Supplementary Fig. 1. Scale (Θ) parameter (A) and shape (α) parameter (B) of a gamma distribution in the five main bioregions. Scale is a measure of the distribution slope and shape is a measure of its skew. Box plot shows the median (center line), first and third quartile (upper and lower hinges) and 1.5 * interquartile range (IQR) (whiskers). Outliers are values beyond 1.5 * IQR. Data from n=78 counties (5 counties were not included because the ecoregion was only represented by 2 or fewer counties).

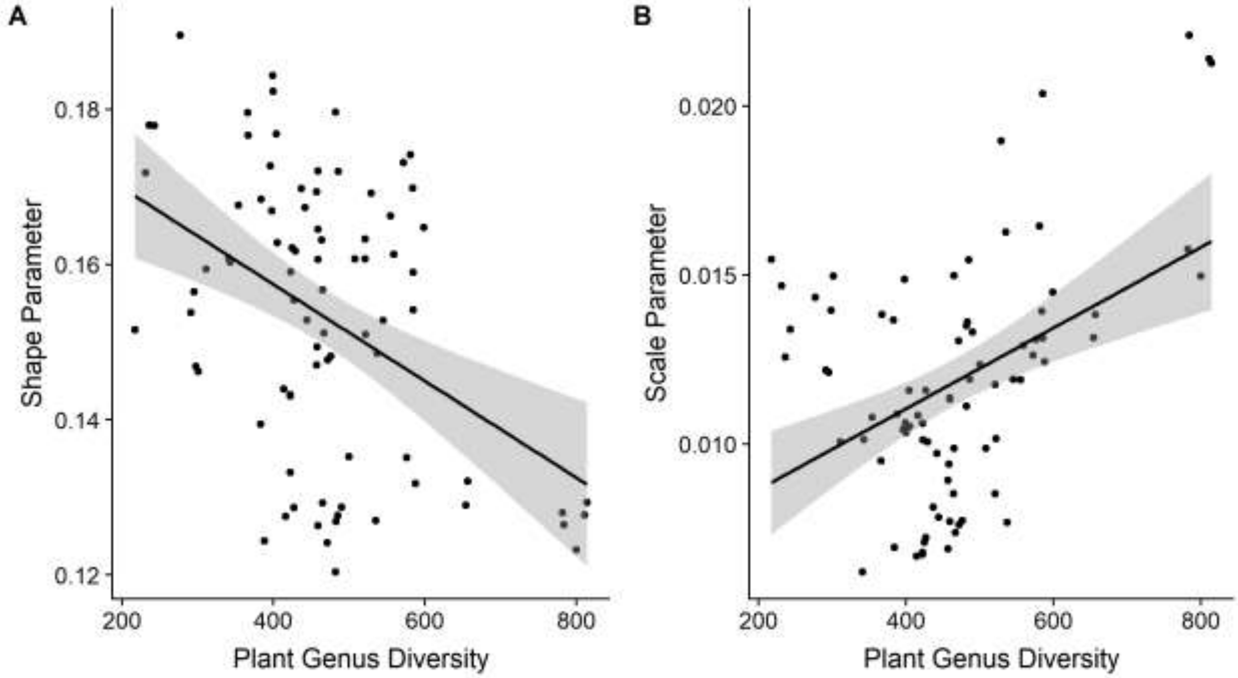

Supplementary Fig. 2. Linear relationship between Shape (α) parameter (A) and Scale (ϴ) parameter (B) over plant genus richness at the county level. Although relationships were significant ($p<0.05$), effect sizes were negligible (Table S1) suggesting no meaningful effect of plant diversity on parameters of the distribution. Line represents the mean predicted relationship and gray ribbon is the 95% confidence interval. Data from n=83 counties.

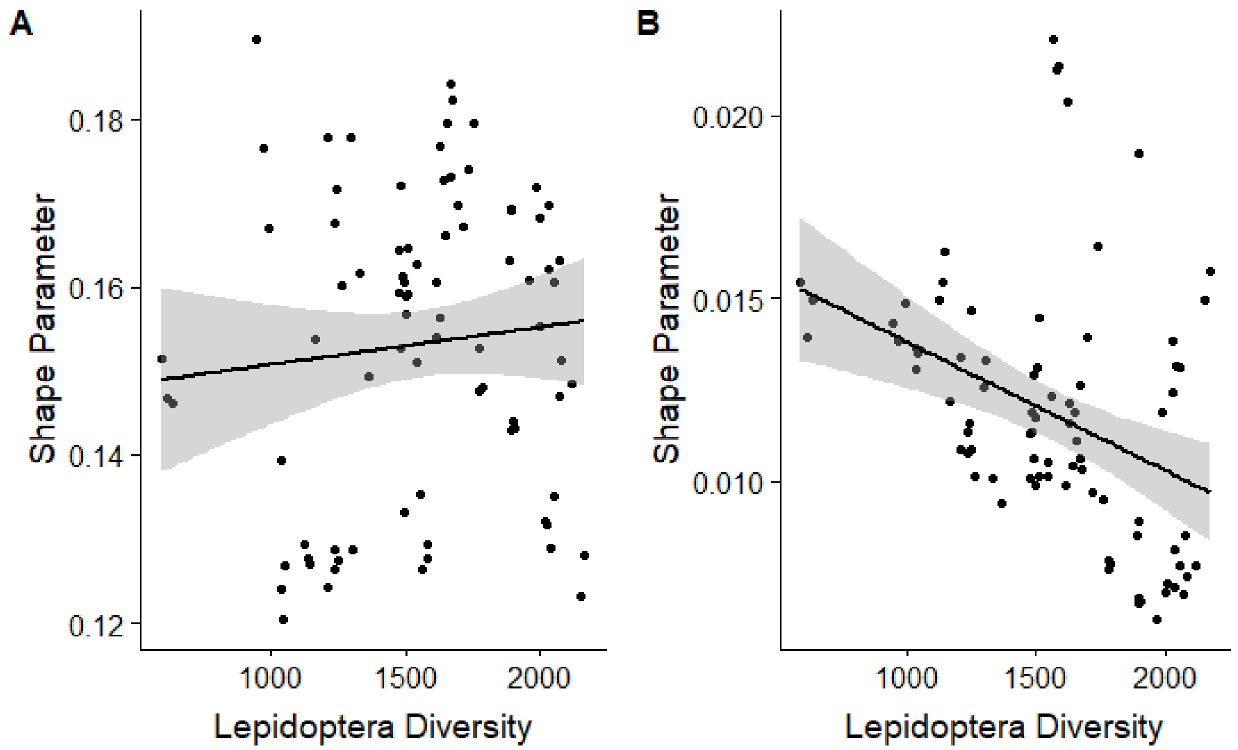

Supplementary Fig. 3. Linear relationship between Shape (α) parameter (A) and Scale (Θ) parameter (B) over Lepidoptera Richness at the county level. Although relationships were significant ($p<0.05$), effect sizes were negligible (Table S1) suggesting no meaningful effect of Lepidoptera diversity on parameters of the distribution. Line represents the mean predicted relationship and gray ribbon is the 95% confidence interval. Data from n=83 counties.

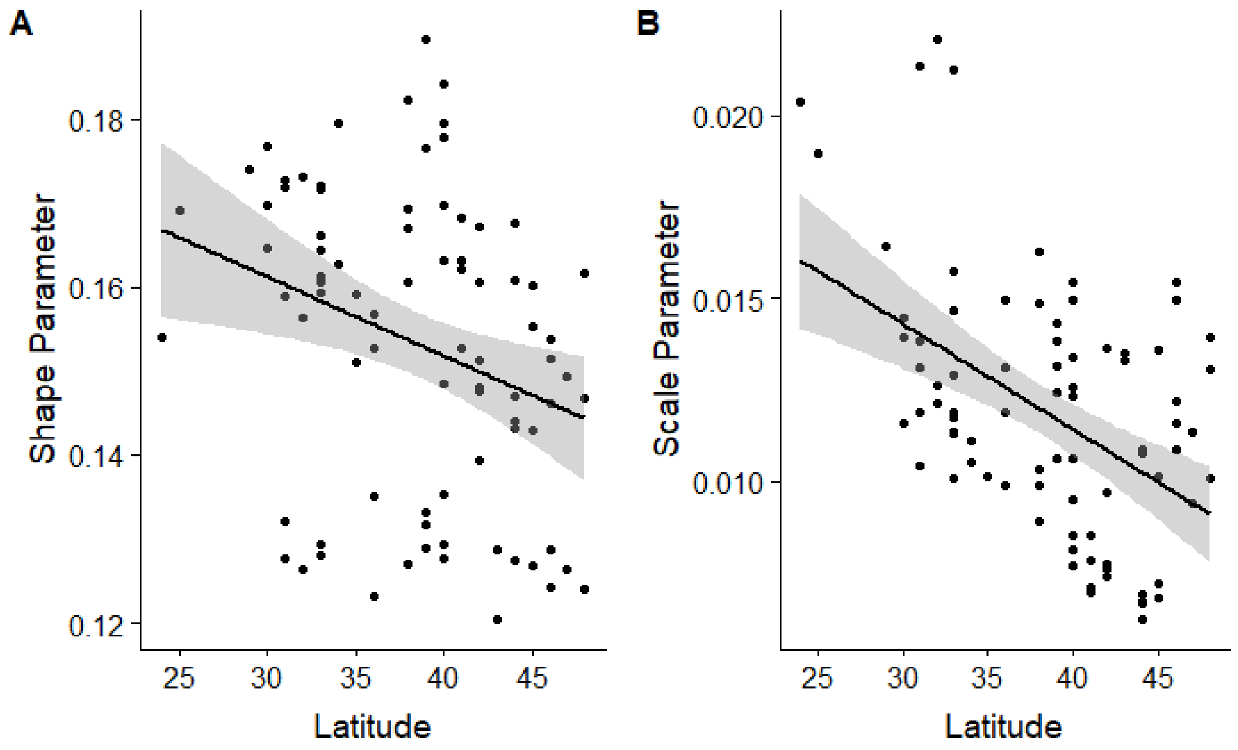

Supplementary Fig. 4. Linear relationship between Shape (α) parameter (A) and scale (Θ) parameter (B) over latitude. Although relationships were significant ($p<0.05$), effect sizes were negligible (Table S1) suggesting no meaningful effect of latitude on parameters of the distribution. Line represents the mean predicted relationship and gray ribbon is the 95% confidence interval. Data from n=83 counties.

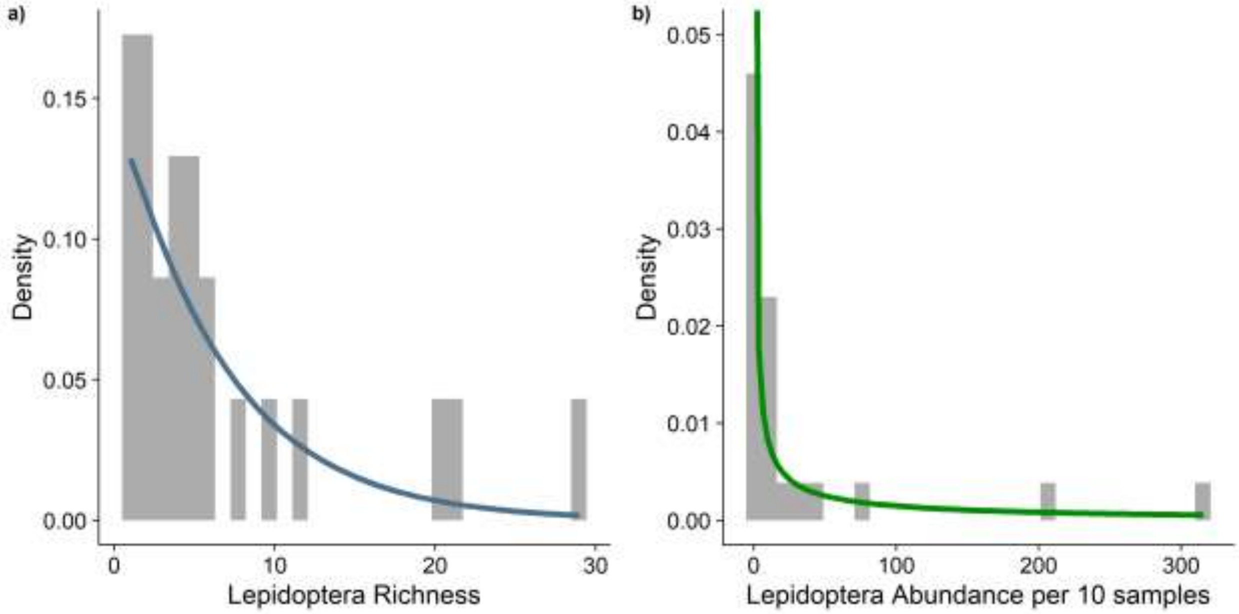

Supplementary Fig. 5. Distribution of a) richness and b) abundance per 10 samples for a field-collected dataset of Lepidoptera on 18 plant genera using standardized methods in hedgerows within New Castle County, DE and Cecil County, MD. See Richard et al.[1] for more details on field methods. Colored lines are fitted gamma distributions to the data.

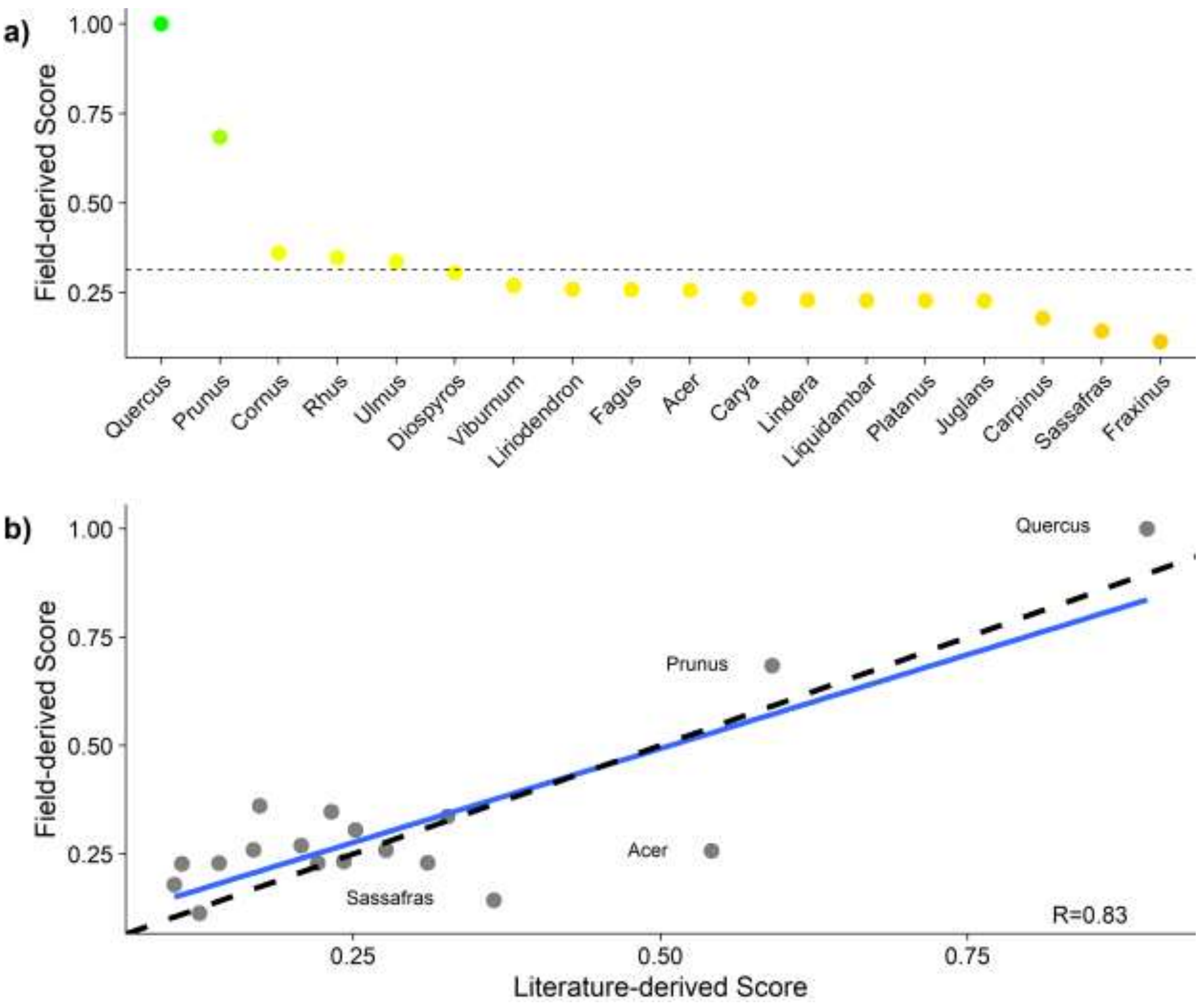

Supplementary Fig. 6. Literature-derived scores and field-derived scores are highly correlated. a) Scores derived from the three-part network analysis using field-derived interaction data systematically collected from woody plants in hedgerows of Northern Delaware. Field data are from Richard et al.[1]. Dashed line is the mean score for the dataset. b) A correlation plot of field-derived scores (x-axis) and literature-derived scores (y-axis). Literature-derived scores are based on host plant data from Sussex, County, Delaware. Blue line is the mean linear slope, and dashed line is a hypothetical slope of 1. Notable tree species are labeled. *Acer* and *Sassafras* trees had lower scores derived from field data than those from the literature. *Prunus* and *Quercus* had the highest scores in either analysis and were identified as keystone species in the county-wide analysis.

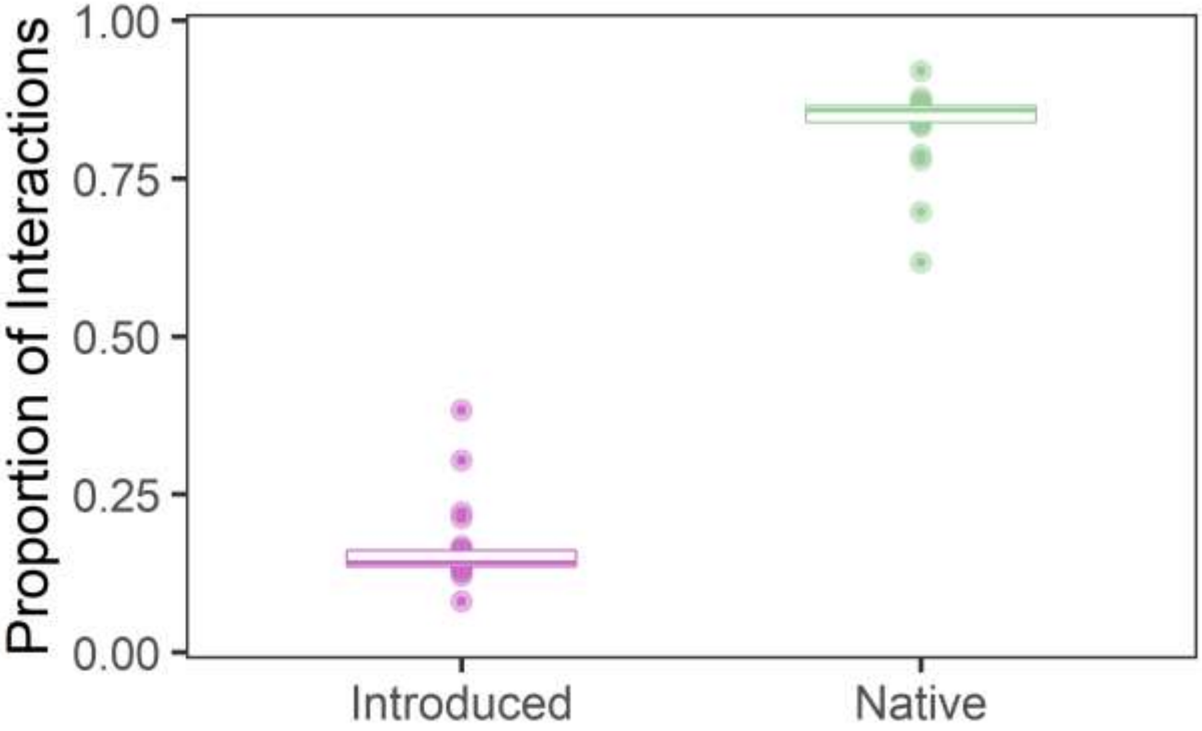

Supplementary Fig. 7. The majority of host plant-Lepidoptera interactions involve native plant genera. Proportions of interactions and species supported by introduced (in purple) and native (in green) plants. Introduced plant genera –Lepidoptera interactions were used to derive the probability of host plant switching by Lepidoptera in simulation models, however, all responses in the models and simulations are based on native plant genera-Lepidoptera interactions. Data from n=83 counties. Box plot shows the median (center line), first and third quartile (upper and lower hinges) and 1.5 * interquartile range (IQR) (whiskers). Outliers are values beyond 1.5 * IQR.

Supplementary Table 1. Statistics for the relationship between the two distribution parameters (shape and scale) and fixed effects that vary by county. Comparisons with ecoregion were made with an analysis of variance test and relationships with plant diversity, Lepidoptera diversity and latitude were made with a linear regression.

†Plant diversity and Lepidoptera diversity models were rescaled such that the β coefficient represents the change with each addition of 100 species. Latitude was rescaled to represent a change in 10 degrees. Count land area was rescaled to represent an addition of 1000 km^2.

Response	Fixed Effect	β ± SE	Rescaled β ± SE†	$F_{4,73}$	t-value	P-value
Shape	Ecoregion			49.38		<0.0001
	Plant Diversity	0.000 ± 0.000	-0.006 ± 0.001		-4.330	<0.0001
	Lepidoptera Diversity	0.000 ± 0.000	0.000 ± 0.001		0.835	0.410
	Latitude	-0.001 ± 0.000	-0.009 ± 0.003		-2.872	0.005
	County Land Area (km^2)	-0.000 ± 0.000	-0.001 ± 0.000		-1.814	0.074
Scale	Ecoregion			22.99		<0.0001
	Plant Diversity	0.000 ± 0.000	0.001 ± 0.000		4.335	<0.0001
	Lepidoptera Diversity	-0.000 ± 0.000	-0.000 ± 0.000		-3.743	0.0003
	Latitude	-0.000 ± 0.000	-0.003 ± 0.001		-4.833	<0.001
	County Land Area (km^2)	-0.000 ± 0.000	-0.000 ± 0.000		-0.520	0.604

Supplementary Table 2. Pairwise comparisons from the analysis of variance test for the difference of means among the 5 ecotypes. Mean difference, 95% confidence intervals and adjusted p-values were calculated using a Tukey's Post-hoc test.

Response	Comparison	Mean Difference	95% Confidence Interval	Adjusted *P*-value
Shape	6-5	-0.025	-0.037, -0.012	<0.0001
	8-5	0.012	0.002, 0.023	0.014
	9-5	0.014	0.002, 0.026	0.018
	10-5	-0.022	-0.035, -0.009	0.0001
	8-6	0.037	0.028, 0.046	<0.0001
	9-6	0.038	0.027, 0.049	<0.0001
	10-6	0.003	-0.010, 0.015	0.978
	9-8	0.001	-0.007, 0.010	0.990
	10-8	-0.034	-0.045, -0.024	<0.0001
	10-9	-0.036	-0.048, -0.024	<0.0001
Scale	6-5	0.005	0.002, 0.008	<0.0001
	8-5	0.003	0.001, 0.005	0.002
	9-5	0.007	0.004, 0.009	<0.0001
	10-5	0.008	0.005, 0.011	<0.0001
	8-6	-0.002	-0.004, -0.000	0.050
	9-6	0.002	-0.001, 0.004	0.308
	10-6	0.003	0.000, 0.006	0.031
	9-8	0.004	0.002, 0.006	<0.0001
	10-8	0.005	0.003, 0.007	<0.0001
	10-9	0.001	-0.001, 0.004	0.678

Supplementary Table 3. Location, plant richness, Lepidoptera richness and summary information for each county included in this analysis.

County & state	Latitude	Longitude	Ecoregion	Native Plants	Native Lepidoptera	Native Lepidoptera that use county plants	Plants for >90% Lepidoptera species	% plants for >90% Lepidoptera species
Barnstable Co. MA	41.7	-70.3	8.5	444	2167	1775	54	12.16
Barrow Co. GA	33.992	-83.72	8.3	311	2365	1475	52	16.72
Beaufort Co. SC	32.431	-80.67	8.5	572	2158	1670	80	13.99
Benson Co. ND	48.071	-99.252	9.2	298	837	618	42	14.09
Berkshire Co. MA	42.45	-73.245	5.3	472	2167	1775	52	11.02
Billings Co. ND	46.914	-103.524	9.3	301	837	637	40	13.29
Blount Co. AL	33.948	-86.273	8.4	559	2095	1488	81	14.46
Boise Co. ID	43.828	-115.834	6.2	482	1565	1046	53	11
Bonner Co. ID	48.276	-116.553	6.2	471	1565	1038	51	11.04
Bradford Co. PA	41.767	-76.443	8.1	425	2588	2030	60	14.12
Brown Co. KS	39.852	-95.536	9.2	367	1346	972	58	15.8
Brown Co. MN	44.314	-94.459	9.2	354	1648	1236	52	14.69
Calhoun Co. MI	42.272	-84.963	8.1	459	2601	2049	69	15.03
Cameron Co. PA	41.511	-78.235	5.3	384	2588	1998	58	15.1
Cameron Co. TX	25.902	-97.497	9.5	529	4378	1895	100	18.9
Cassia Co. ID	42.536	-113.793	10.1	383	1565	1037	47	12.27
Cayuga Co. NY	42.932	-76.566	8.1	467	2635	2078	61	13.07
Charleston Co. GA	30.83	-82.01	8.5	404	2365	1626	69	17.08
Chase Co. KS	38.372	-96.543	9.4	398	1346	993	62	15.58
Clatsop Co. OR	46.188	-123.831	7.1	388	2053	1208	50	12.89
Cleveland Co. AR	33.958	-92.19	8.3	459	2011	1473	68	14.81
Cochise Co. AZ	31.448	-109.928	12.1	811	3715	1585	102	12.58
Colquitt Co. GA	31.18	-83.789	8.3	396	2365	1643	70	17.68
Conecuh Co. AL	31.434	-86.954	8.3	585	2095	1501	86	14.68
Cumberland Co. TN	35.9	-85.023	8.4	522	1981	1544	67	13.03

Custer Co. MT	46.339	-105.761	9.3	291	1866	1167	48	16.49
Delta Co. MI	45.745	-87.064	5.2	427	2601	2001	61	14.29
Desha Co. AR	33.608	-91.207	8.5	459	2011	1480	68	14.75
El Paso Co. TX	31.761	-106.485	10.2	657	4378	2022	94	14.31
Essex Co. MA	42.519	-70.897	8.1	476	2167	1787	54	11.34
Essex Co. NY	44.216	-73.59	5.3	457	2635	2069	60	13.13
Franklin Co. ME	44.67	-70.151	5.3	414	2467	1897	54	13.04
Fulton Co. AR	36.371	-91.823	8.4	545	2011	1486	74	13.58
Gallia Co. OH	38.81	-82.202	8.4	457	2426	1892	70	15.32
Gila Co. AZ	33.394	-110.786	13.1	814	3715	1581	102	12.53
Grand Co. UT	38.57	-109.55	10.1	535	1955	1144	63	11.78
Grant Co. MN	45.999	-96.073	8.1	343	1648	1265	51	14.87
Idaho Co. ID	45.926	-116.122	6.2	483	1566	1054	54	11.18
Inyo Co. CA	36.803	-118.2	10.2	800	3870	2150	104	13
Jackson Co. FL	30.774	-85.227	8.3	584	2883	1694	101	17.29
Jefferson Co. MT	46.236	-112.121	6.2	427	1866	1240	56	13.11
Jewel Co. KS	39.787	-98.21	9.4	277	1346	949	54	19.49
Jo Daviess Co. IL	42.417	-90.429	8.1	442	2235	1713	67	15.16
Jones Co. TX	32.756	-99.896	9.4	295	4378	1626	60	20.4
Knox Co. ME	44.103	-69.109	8.1	422	2467	1904	55	13.03
Lake Co. MN	47.023	-91.671	5.2	458	1648	1363	54	11.79
Lamb Co. TX	33.917	-102.325	9.4	231	4378	1247	51	22.08
Lancaster Co. PA	40.038	-76.305	8.3	521	2588	2070	70	13.44
Laurens Co. SC	34.499	-82.014	8.3	482	2158	1652	76	15.77
Lincoln Co. OR	44.637	-124.053	7.1	416	2053	1253	53	12.74
Logan Co. CO	40.625	-103.208	9.4	236	2716	1300	44	18.64
Malheur Co. OR	43.982	-117.238	10.1	490	2053	1306	61	12.45
Marion Co. IL	38.627	-88.945	8.3	400	2235	1678	67	16.75
McIntosh Co. ND	46.034	-99.371	9.3	217	837	594	38	17.51
McNairy Co. TN	35.17	-88.592	8.3	423	1981	1509	63	14.89
Miami Co. OH	40.039	-84.203	8.2	366	2426	1756	65	17.76

Miller Co. AR	33.442	-94.0377	8.3	521	2011	1499	74	14.2
Mobile Co. AL	30.695	-88.04	8.3	599	2095	1509	85	14.19
Monroe Co. FL	24.555	-81.78	15.4	585	2883	1618	105	17.95
Monterey Co. CA	36.678	-121.655	11.1	576	3870	2054	90	15.63
Nacogdoches Co. TX	31.603	-94.655	8.3	486	4378	1984	92	18.93
Nevada Co. CA	39.261	-121.016	6.2	588	3870	2024	86	14.63
Oscoda Co. MI	44.652	-84.13	5.2	341	2601	1961	55	16.13
Piatt Co. IL	40.027	-88.573	8.2	399	2235	1671	70	17.54
Piscataquis Co. ME	45.183	-69.227	5.3	422	2467	1895	53	12.56
Plumas Co. CA	39.937	-120.947	6.2	654	3870	2037	88	13.61
Rio Blanco Co. CO	40.037	-107.913	10.1	500	2716	1558	64	12.77
Riverside Co. CA	33.953	-117.396	11.1	782	3870	2167	107	13.68
Sanders Co. MT	47.595	-115.337	6.2	459	1866	1238	55	11.98
Suffolk Co. NY	40.917	-72.662	8.5	537	2635	2116	65	12.1
Summit Co. CO	39.481	-106.038	6.2	422	2716	1493	58	13.71
Summit Co. UT	40.918	-111.399	6.2	465	1955	1126	58	12.47
Sumter Co. SC	33.92	-80.341	8.3	555	2158	1649	81	14.59
Sussex Co. DE	38.69	-75.385	8.5	508	1982	1613	51	11.61
Tooele Co. UT	40.531	-112.298	10.1	485	1955	1141	62	12.78
Trumbull Co. OH	41.237	-80.818	8.1	464	2426	1887	69	14.87
Unicoi Co. TN	36.073	-82.518	8.4	465	1981	1500	60	12.9
Union Co. GA	34.876	-83.958	8.4	405	2365	1542	59	15.8
Volusia Co. FL	29.028	-81.303	8.5	581	2883	1735	109	18.76
Washington Co. PA	40.174	-80.246	8.4	437	2588	2033	66	15.1
Wright Co. MN	48.172	-93.875	8.1	429	1648	1333	56	13.05
Yuma Co. AZ	32.693	-114.628	10.2	784	3715	1564	102	13.01
Yuma Co. CO	40.076	-102.223	9.4	243	2716	1212	43	17.7
**AVERAGE**				**467.2**	**2422.35**	**1559.01**	**66.73**	**14.57**

Supplementary Table 4. Attributes for each county included in the distribution analysis. Latitude and longitude were obtained by the location of the county seat. Ecoregion was defined by the Commission for Environmental Cooperation's Ecological Regions of North America map. The shape parameter and scale parameter were obtained using Maximum-likelihood fitting of a Gamma distribution to the data. The *P*-value is from a test of fit for the Gamma distribution. *P*-values > 0.05 indicate a failure to reject the null hypothesis that the data fits a Gamma distribution. Of the 83 counties sampled, 93% (77 counties) failed to reject the null that the data came from a Gamma distribution (p>0.05). Visual inspection of the distributions for the other six counties showed that the overall distribution was similar to Gamma-distributed data (and simulated data with the estimated parameters for the county were also visually similar); therefore, these counties were retained for subsequent analyses. For the Pareto, exponential and log-normal distributions, all counties rejected the null that the data came from these distributions (p<0.05), therefore results are not presented here.

† Key for Ecoregion categories: 5—Northern Forest; 6—Northwestern Forested Mountains; 7—Marine West Coast Forest; 8—Eastern Temperate Forest; 9—Great Plains, 10—North American Deserts; 11—Mediterranean California; 12—Southern Semi-arid Highlands; 15—Tropical Wet Forest. For this analysis, ecoregion 7 was grouped with 6, ecoregion 11 & 12 were grouped with 10 and ecoregion 15 was removed because only one county existed in the dataset.

County	Latitude	Longitude	Ecoregion†	Plant richness	Lepidoptera Richness	Land Area (km^2)	*Gamma Distribution*		
							Shape	Scale	*P*-value
Barnstable Co. MA	41.700	-70.300	8	444	2167	1019.735	0.153	0.008	0.343
Barrow Co. GA	33.992	-83.720	8	311	2365	415.2029	0.159	0.010	0.669
Beaufort Co. SC	32.431	-80.670	8	572	2158	1492.565	0.173	0.013	0.045
Benson Co. ND	48.071	-99.252	9	298	837	3596.759	0.147	0.014	0.355
Berkshire Co. MA	42.450	-73.245	5	472	2167	2400.464	0.148	0.008	0.526
Billings Co. ND	46.914	-103.524	9	301	837	2975.522	0.146	0.015	0.542
Blount Co. AL	33.948	-86.273	8	559	2095	1669.98	0.161	0.013	0.400
Boise Co. ID	43.828	-115.834	6	482	1565	4919.032	0.120	0.014	0.526
Bonner Co. ID	48.276	-116.553	6	471	1565	4492.536	0.124	0.013	0.760
Bradford Co. PA	41.767	-76.443	8	425	2588	2971.766	0.162	0.007	0.709
Brown Co. KS	39.852	-95.536	9	367	1346	1478.553	0.177	0.014	0.926
Brown Co. MN	44.314	-94.459	9	354	1648	1478.553	0.168	0.011	0.156
Calhoun Co. MI	42.272	-84.963	8	459	2601	1829.136	0.161	0.008	0.327
Cameron Co. PA	41.511	-78.235	5	384	2588	1026.236	0.168	0.007	0.697

Cameron Co. TX	25.902	-97.497	9	529	4378	1026.236	0.169	0.019	0.000
Cassia Co. ID	42.536	-113.793	10	383	1565	6643.557	0.139	0.014	0.669
Cayuga Co. NY	42.932	-76.566	8	467	2635	1791.192	0.151	0.007	0.325
Charleston Co. GA	30.830	-82.010	8	404	2365	2003.572	0.177	0.012	0.065
Chase Co. KS	38.372	-96.543	9	398	1346	2002.225	0.167	0.015	0.744
Clatsop Co. OR	46.188	-123.831	7	388	2053	2147.24	0.124	0.011	0.786
Cleveland Co. AR	33.958	-92.190	8	459	2011	1548.25	0.165	0.011	0.339
Cochise Co. AZ	31.448	-109.928	12	811	3715	15969.14	0.128	0.021	0.898
Colquitt Co. GA	31.180	-83.789	8	396	2365	1409.349	0.173	0.010	0.348
Conecuh Co. AL	31.434	-86.954	8	585	2095	2201.914	0.159	0.013	0.357
Cumberland Co. TN	35.900	-85.023	8	522	1981	1763.868	0.151	0.010	0.397
Custer Co. MT	46.339	-105.761	9	291	1866	9798.902	0.154	0.012	0.734
Delta Co. MI	45.745	-87.064	5	427	2601	3033.149	0.155	0.007	0.474
Desha Co. AR	33.608	-91.207	8	459	2011	1989.509	0.172	0.011	0.311
El Paso Co. TX	31.761	-106.485	10	657	4378	2622.867	0.132	0.014	0.008
Essex Co. MA	42.519	-70.897	8	476	2167	1275.73	0.148	0.008	0.452
Essex Co. NY	44.216	-73.590	5	457	2635	1275.73	0.147	0.007	0.599
Franklin Co. ME	44.670	-70.151	5	414	2467	4394.22	0.144	0.007	0.801
Fulton Co. AR	36.371	-91.823	8	545	2011	1601.112	0.153	0.012	0.161
Gallia Co. OH	38.810	-82.202	8	457	2426	1208.313	0.169	0.009	0.258
Gila Co. AZ	33.394	-110.786	12	814	3715	12323.04	0.129	0.021	0.885
Grand Co. UT	38.570	-109.550	10	535	1955	9509.289	0.127	0.016	0.917
Grant Co. MN	45.999	-96.073	8	343	1648	1419.734	0.160	0.010	0.145
Idaho Co. ID	45.926	-116.122	6	483	1566	21956.34	0.127	0.014	0.609
Inyo Co. CA	36.803	-118.200	10	800	3870	26368.48	0.123	0.015	0.052
Jackson Co. FL	30.774	-85.227	8	584	2883	2376.998	0.170	0.014	0.319
Jefferson Co. MT	46.236	-112.121	6	427	1866	4289.713	0.129	0.012	0.636
Jewel Co. KS	39.787	-98.210	9	277	1346	2356.33	0.190	0.014	0.750
Jo Daviess Co. IL	42.417	-90.429	8	442	2235	1556.823	0.167	0.010	0.179
Jones Co. TX	32.756	-99.896	9	295	4378	2404.945	0.156	0.012	0.025
Knox Co. ME	44.103	-69.109	8	422	2467	945.6867	0.143	0.007	0.786

Lake Co. MN	47.023	-91.671	5	458	1648	5463.061	0.149	0.009	0.365
Lamb Co. TX	33.917	-102.325	9	231	4378	2631.906	0.172	0.015	0.938
Lancaster Co. PA	40.038	-76.305	8	521	2588	2444.468	0.163	0.009	0.113
Laurens Co. SC	34.499	-82.014	8	482	2158	1848.742	0.180	0.011	0.353
Lincoln Co. OR	44.637	-124.053	7	416	2053	2537.423	0.128	0.011	0.887
Logan Co. CO	40.625	-103.208	9	236	2716	4761.845	0.178	0.013	0.328
Malheur Co. OR	43.982	-117.238	10	490	2053	25608.7	0.129	0.013	0.290
Marion Co. IL	38.627	-88.945	8	400	2235	1482.257	0.182	0.010	0.076
McIntosh Co. ND	46.034	-99.371	9	217	837	2524.551	0.152	0.015	0.629
McNairy Co. TN	35.170	-88.592	8	423	1981	1457.807	0.159	0.010	0.353
Miami Co. OH	40.039	-84.203	8	366	2426	1053.042	0.180	0.010	0.112
Miller Co. AR	33.442	-94.038	8	521	2011	1620.252	0.161	0.012	0.220
Mobile Co. AL	30.695	-88.040	8	599	2095	3184.25	0.165	0.014	0.096
Monroe Co. FL	24.555	-81.780	15	585	2883	2546.695	0.154	0.020	0.000
Monterey Co. CA	36.678	-121.655	11	576	3870	8496.754	0.135	0.013	0.396
Nacogdoches Co. TX	31.603	-94.655	8	486	4378	2451.539	0.172	0.012	0.381
Nevada Co. CA	39.261	-121.016	6	588	3870	2480.624	0.132	0.012	0.523
Oscoda Co. MI	44.652	-84.130	5	341	2601	1465.241	0.161	0.006	0.913
Piatt Co. IL	40.027	-88.573	8	399	2235	1137.528	0.184	0.011	0.096
Piscataquis Co. ME	45.183	-69.227	5	422	2467	10258.63	0.143	0.007	0.714
Plumas Co. CA	39.937	-120.947	6	654	3870	6612.374	0.129	0.013	0.344
Rio Blanco Co. CO	40.037	-107.913	10	500	2716	8342.209	0.135	0.012	0.409
Riverside Co. CA	33.953	-117.396	11	782	3870	18664.78	0.128	0.016	0.033
Sanders Co. MT	47.595	-115.337	6	459	1866	7149.747	0.126	0.011	0.850
Suffolk Co. NY	40.917	-72.662	8	537	2635	2362.21	0.149	0.008	0.214
Summit Co. CO	39.481	-106.038	6	422	2716	1575.652	0.133	0.011	0.694
Summit Co. UT	40.918	-111.399	6	465	1955	1575.652	0.129	0.015	0.686
Sumter Co. SC	33.920	-80.341	8	555	2158	1722.531	0.166	0.012	0.117
Sussex Co. DE	38.690	-75.385	8	508	1982	2424.447	0.161	0.010	0.162
Tooele Co. UT	40.531	-112.298	10	485	1955	17978.1	0.128	0.015	0.814
Trumbull Co. OH	41.237	-80.818	8	464	2426	1601.397	0.163	0.009	0.472

Unicoi Co. TN	36.073	-82.518	8	465	1981	482.1803	0.157	0.010	0.538
Union Co. GA	34.876	-83.958	8	405	2365	833.7987	0.163	0.011	0.243
Volusia Co. FL	29.028	-81.303	8	581	2883	2851.668	0.174	0.016	0.052
Washington Co. PA	40.174	-80.246	8	437	2588	2219.604	0.170	0.008	0.315
Wright Co. MN	48.172	-93.875	8	429	1648	1713.181	0.162	0.010	0.095
Yuma Co. AZ	32.693	-114.628	10	784	3715	14281.23	0.126	0.022	0.562
Yuma Co. CO	40.076	-102.223	9	243	2716	14281.23	0.178	0.013	0.222

Supplementary Table 5. Mean importance score for 288 woody plant genera across 25 counties in the United States in descending order from largest to smallest. Herbaceous plants were not included in this analysis.

Rank	Plant Genus	Mean Importance Score	# counties in dataset
1	*Quercus*	0.788 ± 0.132	23
2	*Salix*	0.552 ± 0.157	25
3	*Prunus*	0.51 ± 0.11	25
4	*Pinus*	0.464 ± 0.113	22
5	*Populus*	0.438 ± 0.154	23
6	*Betula*	0.389 ± 0.124	20
7	*Vaccinium*	0.373 ± 0.159	21
8	*Acer*	0.369 ± 0.131	25
9	*Carya*	0.367 ± 0.101	17
10	*Malus*	0.336 ± 0.082	12
11	*Ulmus*	0.323 ± 0.084	18
12	*Alnus*	0.305 ± 0.12	23
13	*Sequoia*	0.294 ± NA	1
14	*Tilia*	0.288 ± 0.083	19
15	*Prosopis*	0.284 ± NA	1
16	*Crataegus*	0.282 ± 0.102	23
17	*Rubus*	0.28 ± 0.099	24
18	*Menziesia*	0.279 ± 0.018	2
19	*Castanea*	0.278 ± 0.068	15
20	*Picea*	0.276 ± 0.133	7
21	*Pseudotsuga*	0.274 ± 0.17	6
22	*Fraxinus*	0.274 ± 0.128	22
23	*Adenostoma*	0.271 ± 0.029	2
24	*Chrysolepis*	0.265 ± 0.042	2
25	*Viburnum*	0.263 ± 0.11	20
26	*Ribes*	0.26 ± 0.137	19
27	*Corylus*	0.259 ± 0.106	18
28	*Myrica*	0.258 ± 0.056	6
29	*Vitis*	0.255 ± 0.12	21
30	*Elaeagnus*	0.251 ± NA	1
31	*Rosa*	0.248 ± 0.083	24
32	*Ceanothus*	0.243 ± 0.14	24
33	*Callicarpa*	0.242 ± 0.092	7
34	*Arctostaphylos*	0.24 ± 0.11	12
35	*Parkinsonia*	0.238 ± NA	1
36	*Cylindropuntia*	0.238 ± NA	1
37	*Hamamelis*	0.237 ± 0.077	16
38	*Arbutus*	0.237 ± 0.065	2

39	*Fagus*	0.237 ± 0.106	15
40	*Calocedrus*	0.237 ± 0.001	2
41	*Spiraea*	0.236 ± 0.078	12
42	*Robinia*	0.235 ± 0.092	8
43	*Encelia*	0.235 ± NA	1
44	*Tsuga*	0.234 ± 0.073	13
45	*Lycium*	0.232 ± NA	1
46	*Sabal*	0.231 ± 0.047	6
47	*Dodonaea*	0.23 ± NA	1
48	*Juniperus*	0.23 ± 0.111	25
49	*Hydrangea*	0.23 ± 0.033	5
50	*Fouquieria*	0.229 ± NA	1
51	*Juglans*	0.229 ± 0.102	17
52	*Ostrya*	0.228 ± 0.114	16
53	*Simmondsia*	0.228 ± NA	1
54	*Abies*	0.228 ± 0.097	10
55	*Frangula*	0.227 ± 0.061	9
56	*Sebastiania*	0.222 ± NA	1
57	*Zanthoxylum*	0.222 ± 0.071	10
58	*Pinckneya*	0.222 ± 0.131	2
59	*Rhus*	0.222 ± 0.083	23
60	*Cornus*	0.221 ± 0.085	25
61	*Comptonia*	0.221 ± 0.119	8
62	*Heteromeles*	0.219 ± NA	1
63	*Symphoricarpos*	0.218 ± 0.09	20
64	*Amelanchier*	0.217 ± 0.101	24
65	*Nolina*	0.217 ± NA	1
66	*Smilax*	0.217 ± 0.073	20
67	*Gymnocladus*	0.217 ± 0.052	5
68	*Taxodium*	0.217 ± 0.089	8
69	*Sapindus*	0.217 ± 0.133	2
70	*Illicium*	0.216 ± 0.042	2
71	*Rhamnus*	0.216 ± 0.052	10
72	*Liquidambar*	0.216 ± 0.046	11
73	*Malosma*	0.213 ± NA	1
74	*Larix*	0.209 ± 0.076	6
75	*Ampelopsis*	0.209 ± 0.105	6
76	*Eriodictyon*	0.208 ± 0.032	2
77	*Umbellularia*	0.208 ± 0.019	2
78	*Celtis*	0.206 ± 0.084	20
79	*Condalia*	0.204 ± NA	1
80	*Sophora*	0.204 ± NA	1
81	*Chamaedaphne*	0.203 ± 0.068	8

82	*Taxus*	0.203 ± 0.034	8
83	*Sassafras*	0.202 ± 0.066	13
84	*Peniocereus*	0.202 ± NA	1
85	*Diospyros*	0.201 ± 0.088	12
86	*Lonicera*	0.201 ± 0.079	21
87	*Gleditsia*	0.201 ± 0.067	12
88	*Thuja*	0.201 ± 0.101	10
89	*Andromeda*	0.2 ± 0.03	5
90	*Decodon*	0.199 ± 0.098	9
91	*Chilopsis*	0.198 ± NA	1
92	*Toxicodendron*	0.198 ± 0.073	23
93	*Parthenocissus*	0.197 ± 0.088	20
94	*Wisteria*	0.196 ± 0.121	4
95	*Chamaecyparis*	0.196 ± 0.098	7
96	*Choisya*	0.196 ± NA	1
97	*Larrea*	0.196 ± NA	1
98	*Cephalanthus*	0.195 ± 0.082	18
99	*Celastrus*	0.195 ± 0.037	9
100	*Dirca*	0.195 ± 0.032	12
101	*Magnolia*	0.194 ± 0.108	12
102	*Ferocactus*	0.194 ± NA	1
103	*Lyonia*	0.194 ± 0.073	14
104	*Berberis*	0.193 ± 0.014	3
105	*Leucophyllum*	0.193 ± NA	1
106	*Cotinus*	0.193 ± 0.003	2
107	*Sorbus*	0.193 ± 0.067	5
108	*Cercis*	0.192 ± 0.057	13
109	*Lantana*	0.192 ± NA	1
110	*Staphylea*	0.191 ± 0.054	11
111	*Tecoma*	0.191 ± NA	1
112	*Nemopanthus*	0.19 ± 0.113	6
113	*Muhlenbergia*	0.19 ± 0.08	24
114	*Erythrina*	0.189 ± 0.011	3
115	*Ilex*	0.188 ± 0.059	16
116	*Nyssa*	0.187 ± 0.072	13
117	*Platanus*	0.186 ± 0.069	17
118	*Vauquelinia*	0.186 ± NA	1
119	*Sambucus*	0.185 ± 0.097	25
120	*Malacothamnus*	0.184 ± NA	1
121	*Campsis*	0.182 ± 0.055	12
122	*Lindera*	0.182 ± 0.055	14
123	*Oemleria*	0.18 ± 0.056	2
124	*Morus*	0.179 ± 0.06	13

125	*Symplocos*	0.178 ± 0.054	8
126	*Diervilla*	0.178 ± 0.054	9
127	*Kalmia*	0.176 ± 0.072	16
128	*Holodiscus*	0.176 ± 0.168	6
129	*Hazardia*	0.176 ± NA	1
130	*Liriodendron*	0.175 ± 0.055	12
131	*Catalpa*	0.172 ± 0.062	5
132	*Lepechinia*	0.172 ± NA	1
133	*Chionanthus*	0.171 ± 0.043	10
134	*Persea*	0.171 ± 0.085	6
135	*Physocarpus*	0.171 ± 0.063	13
136	*Carpinus*	0.17 ± 0.091	16
137	*Stewartia*	0.169 ± 0.057	5
138	*Asimina*	0.167 ± 0.094	13
139	*Litsea*	0.164 ± 0.143	3
140	*Porophyllum*	0.164 ± 0.006	2
141	*Aesculus*	0.162 ± 0.076	10
142	*Aronia*	0.162 ± 0.069	16
143	*Fremontodendron*	0.161 ± 0.02	2
144	*Eysenhardtia*	0.161 ± NA	1
145	*Olneya*	0.161 ± NA	1
146	*Ptelea*	0.16 ± 0.075	11
147	*Lysiloma*	0.16 ± NA	1
148	*Purshia*	0.159 ± 0.069	5
149	*Gaylussacia*	0.158 ± 0.062	14
150	*Keckiella*	0.158 ± 0.063	2
151	*Halesia*	0.158 ± 0.057	5
152	*Morella*	0.158 ± 0.072	14
153	*Leucothoe*	0.157 ± 0.111	5
154	*Bebbia*	0.157 ± NA	1
155	*Clethra*	0.156 ± 0.081	9
156	*Euonymus*	0.155 ± 0.072	14
157	*Pieris*	0.154 ± 0.01	2
158	*Chamaebatiaria*	0.152 ± 0.077	3
159	*Oxydendrum*	0.151 ± 0.076	8
160	*Carnegiea*	0.15 ± NA	1
161	*Gordonia*	0.149 ± 0.015	2
162	*Philadelphus*	0.148 ± 0.075	9
163	*Ziziphus*	0.147 ± NA	1
164	*Cercocarpus*	0.144 ± 0.084	5
165	*Fallugia*	0.144 ± NA	1
166	*Shepherdia*	0.144 ± 0.105	9
167	*Rhododendron*	0.143 ± 0.066	19

168	*Gaultheria*	0.143 ± 0.09	15
169	*Eubotrys*	0.142 ± 0.072	9
170	*Aloysia*	0.142 ± NA	1
171	*Maclura*	0.14 ± 0.134	2
172	*Dasylirion*	0.138 ± NA	1
173	*Bignonia*	0.138 ± 0.084	9
174	*Sarcobatus*	0.137 ± 0.11	5
175	*Cordia*	0.134 ± NA	1
176	*Calycanthus*	0.131 ± 0.019	5
177	*Coryphantha*	0.128 ± NA	1
178	*Sideroxylon*	0.127 ± 0.082	9
179	*Serenoa*	0.127 ± 0.172	2
180	*Berchemia*	0.123 ± 0.092	7
181	*Empetrum*	0.12 ± 0.169	2
182	*Cissus*	0.117 ± 0.086	4
183	*Calliandra*	0.106 ± NA	1
184	*Phoradendron*	0.1 ± 0.101	14
185	*Buddleja*	0.099 ± NA	1
186	*Echinocactus*	0.099 ± NA	1
187	*Ephedra*	0.095 ± 0.1	2
188	*Nemophila*	0.085 ± 0.103	9
189	*Krameria*	0.078 ± 0.11	2
190	*Forestiera*	0.07 ± 0.097	5
191	*Arceuthobium*	0.068 ± 0.097	9
192	*Echinocereus*	0.059 ± 0.102	3
193	*Garrya*	0.042 ± 0.072	3
194	*Lorandersonia*	0.026 ± NA	1
195	*Dryas*	0.026 ± NA	1
196	*Grayia*	0.022 ± 0.004	2
197	*Piptochaetium*	0.015 ± 0.047	13
198	*Krascheninnikovia*	0.007 ± 0.014	4
199	*Acaciella*	0 ± 0	2
200	*Adlumia*	0 ± 0	6
201	*Allenrolfea*	0 ± 0	3
202	*Arctous*	0 ± NA	1
203	*Aspicarpa*	0 ± NA	1
204	*Atamisquea*	0 ± NA	1
205	*Ayenia*	0 ± NA	1
206	*Barkleyanthus*	0 ± NA	1
207	*Bernardia*	0 ± NA	1
208	*Borrichia*	0 ± 0	2
209	*Bouvardia*	0 ± NA	1
210	*Bursera*	0 ± NA	1

211	*Callaeum*	0 ± NA	1
212	*Calycocarpum*	0 ± 0	3
213	*Canotia*	0 ± NA	1
214	*Cartrema*	0 ± 0	2
215	*Cassiope*	0 ± NA	1
216	*Castela*	0 ± NA	1
217	*Chamaebatia*	0 ± NA	1
218	*Chrysoma*	0 ± NA	1
219	*Cladrastis*	0 ± 0	2
220	*Cliftonia*	0 ± 0	2
221	*Cocculus*	0 ± 0	7
222	*Colubrina*	0 ± NA	1
223	*Crocanthemum*	0 ± 0	18
224	*Crossosoma*	0 ± NA	1
225	*Dendromecon*	0 ± NA	1
226	*Diphasiastrum*	0 ± 0	13
227	*Diplacus*	0 ± 0	3
228	*Ditrysinia*	0 ± 0	3
229	*Echinomastus*	0 ± NA	1
230	*Elliottia*	0 ± NA	1
231	*Escobaria*	0 ± 0	3
232	*Fagonia*	0 ± NA	1
233	*Fallopia*	0 ± 0	14
234	*Fendlera*	0 ± NA	1
235	*Fendlerella*	0 ± NA	1
236	*Fothergilla*	0 ± 0	2
237	*Glossopetalon*	0 ± NA	1
238	*Grusonia*	0 ± 0	2
239	*Haplophyton*	0 ± NA	1
240	*Hedyotis*	0 ± NA	1
241	*Hesperocyparis*	0 ± 0	2
242	*Horsfordia*	0 ± NA	1
243	*Isotrema*	0 ± 0	6
244	*Jamesia*	0 ± 0	3
245	*Johnstonella*	0 ± 0	2
246	*Koanophyllon*	0 ± NA	1
247	*Koeberlinia*	0 ± NA	1
248	*Leitneria*	0 ± NA	1
249	*Licania*	0 ± 0	2
250	*Loeselia*	0 ± NA	1
251	*Loiseleuria*	0 ± NA	1
252	*Mammillaria*	0 ± NA	1
253	*Mariosousa*	0 ± NA	1

254	*Menispermum*	0 ± 0	13
255	*Mortonia*	0 ± NA	1
256	*Nestronia*	0 ± 0	2
257	*Neviusia*	0 ± NA	1
258	*Nothochelone*	0 ± NA	1
259	*Notholithocarpus*	0 ± NA	1
260	*Oplopanax*	0 ± NA	1
261	*Pediocactus*	0 ± 0	3
262	*Peraphyllum*	0 ± 0	2
263	*Petalonyx*	0 ± NA	1
264	*Petrophytum*	0 ± 0	2
265	*Peucephyllum*	0 ± NA	1
266	*Phyllanthopsis*	0 ± NA	1
267	*Phyllodoce*	0 ± 0	2
268	*Pickeringia*	0 ± NA	1
269	*Picrothamnus*	0 ± 0	2
270	*Pisonia*	0 ± NA	1
271	*Planera*	0 ± 0	4
272	*Pleurocoronis*	0 ± NA	1
273	*Psorothamnus*	0 ± NA	1
274	*Rhapidophyllum*	0 ± 0	2
275	*Romneya*	0 ± NA	1
276	*Sageretia*	0 ± 0	2
277	*Salazaria*	0 ± NA	1
278	*Schaefferia*	0 ± NA	1
279	*Schisandra*	0 ± 0	2
280	*Sclerocactus*	0 ± NA	1
281	*Senegalia*	0 ± NA	1
282	*Sequoiadendron*	0 ± NA	1
283	*Tetracoccus*	0 ± NA	1
284	*Torreya*	0 ± 0	2
285	*Vachellia*	0 ± NA	1
286	*Washingtonia*	0 ± NA	1
287	*Whipplea*	0 ± NA	1
288	*Zapoteca*	0 ± NA	1

REFERENCES

1. Richard, M., Tallamy, D.W. and Mitchell, A.B. Introduced plants reduce species interactions. Biol. Invas. 21(3), 983-992 (2019).
