# Supplementary material for: Few keystone plant genera support the majority of Lepidoptera species
Source: Nat Commun. 2020 Nov 13;11:5751. doi: 10.1038/s41467-020-19565-4 (PMC7666120; doi:10.1038/s41467-020-19565-4)
Supplement: Supplementary file 3 — Description of Additional Supplementary Files [file 41467_2020_19565_MOESM3_ESM.pdf]

Description of Additional Supplementary Files

File Name: Supplementary Data 1
Description: County data

File Name: Supplementary Data 2
Description: Lepidoptera richness for each plant genera in each county

File Name: Supplementary Data 3
Description: R Code for network analyses

File Name: Supplementary Data 4
Description: R Code for restoration simulation with woody plants

File Name: Supplementary Data 5
Description: R Code for restoration simulation with herbaceous plants

File Name: Supplementary Data 6
Description: Word document of entire Lepidoptera bibliography used to create the interaction dataset.
